# Single breath-hold saturation recovery 3D cardiac T1 mapping via compressed SENSE at 3T

**DOI:** 10.1007/s10334-020-00848-2

**Published:** 2020-05-14

**Authors:** Tiago Ferreira da Silva, Carlos Galan-Arriola, Paula Montesinos, Gonzalo Javier López-Martín, Manuel Desco, Valentín Fuster, Borja Ibáñez, Javier Sanchez-Gonzalez

**Affiliations:** 1 Philips Healthcare Iberia, Madrid, Spain; 2 Centro Nacional de Investigaciones Cardiovasculares Carlos III (CNIC), Madrid, Spain; 3 CIBER de Enfermedades Cardiovasculares (CIBERCV), Madrid, Spain; 4 Unidad de imagen avanzada, Centro Nacional de Investigaciones Cardiovasculares Carlos III (CNIC), Madrid, Spain; 5 CIBER de salud mental (CIBERSAM), Madrid, Spain; 6 Departamento de Bioingeniería e Ingeniería Aerospacial, Universidad Carlos III, Madrid, Spain; 7 Medicina y Cirugía Experimental, Instituto de Investigacion Sanitaria Gregorio Marañón, Madrid, Spain; 8 The Zena and Michael A. Wiener CVI, Icahn School of Medicine at Mount Sinai, New York, USA; 9 IIS-Fundación Jiménez Díaz University Hospital, Madrid, Spain

**Keywords:** Cardiovascular magnetic resonance, Tissue characterization, 3D cardiac T1 mapping, Single breath-hold, Saturation recovery

## Abstract

**Objectives:**

To propose and validate a novel imaging sequence that uses a single breath-hold whole-heart 3D T1 saturation recovery compressed SENSE rapid acquisition (SACORA) at 3T.

**Methods:**

The proposed sequence combines flexible saturation time sampling, compressed SENSE, and sharing of saturation pulses between two readouts acquired at different RR intervals. The sequence was compared with a 3D saturation recovery single-shot acquisition (SASHA) implementation with phantom and in vivo experiments (pre and post contrast; 7 pigs) and was validated against the reference inversion recovery spin echo (IR-SE) sequence in phantom experiments.

**Results:**

Phantom experiments showed that the T1 maps acquired by 3D SACORA and 3D SASHA agree well with IR-SE. In vivo experiments showed that the pre-contrast and post-contrast T1 maps acquired by 3D SACORA are comparable to the corresponding 3D SASHA maps, despite the shorter acquisition time (15s vs. 188s, for a heart rate of 60 bpm). Mean septal pre-contrast T1 was 1453 ± 44 ms with 3D SACORA and 1460 ± 60 ms with 3D SASHA. Mean septal post-contrast T1 was 824 ± 66 ms and 824 ± 60 ms.

**Conclusion:**

3D SACORA acquires 3D T1 maps in 15 heart beats (heart rate, 60 bpm) at 3T. In addition to its short acquisition time, the sequence achieves good T1 estimation precision and accuracy.

**Electronic supplementary material:**

The online version of this article (10.1007/s10334-020-00848-2) contains supplementary material, which is available to authorized users.

## Introduction

Cardiovascular magnetic resonance (CMR) is a medical imaging modality whose wide applications include tissue characterization across different physiological and pathological conditions. CMR longitudinal relaxation time (T1) mapping has become particularly relevant since it differs between healthy and diseased tissue, and aims to play an important role in clinical decision-making in cardiovascular disease [1–3]. T1 mapping provides pixel-wise T1 values by fitting images acquired during the T1 magnetization recovery at different times after application of an inversion pulse [4], a saturation pulse [5], or a combination of both [6].

CMR T1 mapping is complex due to the heart’s motion, and several two-dimensional (2D) pulse sequences have been presented in recent years to tackle the problem [7–9]. However, these sequences are designed to acquire only a single slice per breath-hold. The modified Look-Locker inversion recovery (MOLLI) sequence applies an inversion pulse followed by different single-shot steady-state free precession (SSFP) readouts over multiple heartbeats [4]. MOLLI is precise and reproducible; however, it underestimates T1, mainly due to the magnetization transfer effect and imperfect inversion efficiency [8]. To overcome this limitation, saturation recovery single-shot acquisition (SASHA) replaces the inversion pulses with saturation pulses, thus avoiding the underestimation of T1 values due to incomplete recovery of the signal after the inversion pulse [5]. This approach improves accuracy at the cost of a lower signal–noise ratio (SNR). A different 2D saturation recovery sequence design is provided by modified Look–Locker acquisition using saturation recovery (MLLSR), which allows the saturation pulses to be shared between several readouts acquired in different heart beats to minimize T1 estimation error and provide high flexibility [10]. Lastly, saturation pulse-prepared heart rate independent inversion recovery (SAPPHIRE) is a hybrid approach that uses inversion and saturation pulses and sits at the midpoint between the advantages and limitations of saturation and inversion schemes [6].

Highly accurate T1 estimates can also be obtained with three-dimensional (3D) pulse sequences based on saturation recovery and developed to fully cover the left ventricle (LV). 3D SASHA combines a 2D SASHA-based pulse scheme with free-breathing imaging for 3D acquisition at 1.5T [11]. A more recent free-breathing 3D T1 mapping sequence provides 3D acquisition at 3T, based on a new pulse scheme that acquires substantially fewer T1-weighted images than 3D SASHA [12]. These 3D saturation recovery approaches offer higher SNR and good spatial coverage at the cost of longer acquisition times. They require navigator-triggered free-breathing and, therefore, rely on the respiratory navigation performance to achieve good image quality and acceptable acquisition time. Acquisition time is nevertheless in the range of minutes, even when denoising and optimization techniques are used [13, 14]. Such long acquisition time compromises the feasibility of T1 mapping during contrast equilibrium and reduce clinical applicability. Furthermore, these 3D saturation recovery sequences acquire all T1-weighted images in the same RR interval of the saturation pulse, which compromises T1 estimation quality, particularly for long T1s and high heart rates [15].

Independent of magnetic preparation, most proposed T1 mapping techniques are acquired using SSFP readout techniques. At 3T, SSFP sequences have been associated with higher energy deposition and increased off-resonance artifacts [16]. Alternatives to SSFP sequences are spoiled sequences. These sequences use fast low-angle shot (FLASH) imaging readouts to avoid off-resonance artifacts and to eliminate transverse relaxation time (T2) dependence [12, 17, 18]. The main limitation of FLASH schemes is the low SNR compared with SSFP sequences.

In addition to the development of pulse sequences, new techniques have emerged to accelerate acquisition. Compressed sensing exploits the sparsity or compressibility of CMR images and accelerates acquisition by undersampling without significantly degrading images [19]. This technique has been successfully used to accelerate 3D cardiac imaging [20–22]. Compressed SENSE is built on compressed sensing and incorporates components of the parallel acquisition technique SENSE [23]. Studies showing the feasibility of compressed SENSE have recently been published for T1 calculation in the brain [24] and for cardiac cine imaging [25]. As expected, the method significantly reduced acquisition time, particularly in 3D acquisitions.

In this study, we propose and validate the 3D saturation recovery compressed SENSE rapid acquisition (3D SACORA) imaging sequence, a new 3D T1 spoiled saturation recovery mapping technique for acquisition of the entire LV in a single breath-hold at 3T. The proposed technique combines flexible saturation time sampling, compressed SENSE, and sharing of the saturation pulses between two readouts acquired at different RR intervals. This approach aims to achieve good quality single breath-hold saturation recovery 3D T1 mapping and stability over a wide range of heart rates (HRs).

## Methods

### Pulse sequence design

**Fig. 1:**
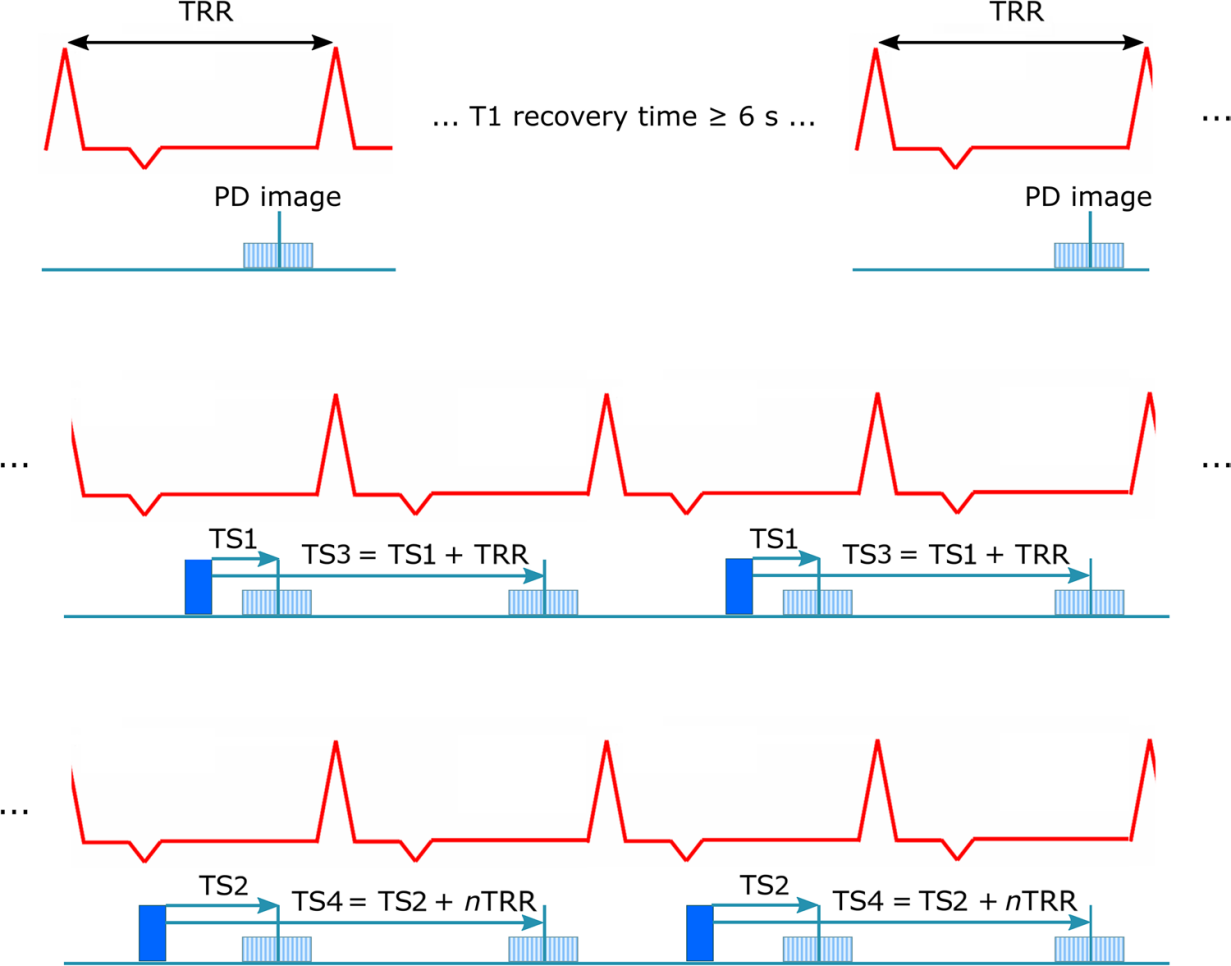
3D SACORA pulse sequence. The sequence performs two turbo field echo (TFE) shots for each 3D image: in the PD image block (top), the two TFEs are separated by a minimum 6 s magnetization recovery; two images are acquired at TS1 and TS3 (middle row); and two images are acquired at TS2 and TS4 (bottom row). TS1 = 250 ms, TS2 = 500 ms (if allowed by the HR), TS3 = TS1 + RR interval, TS4 = TS2 + *n*RR intervals (*n* depending on HR)

3D SACORA consists of three distinct blocks (Fig. 1). The sequence begins with proton density (PD) images to avoid T1 effects from prior saturation pulses (first block). Full signal recovery is ensured by waiting a minimum of 6 s between the PD readouts [12, 26]. The second block consists of images acquired at saturation time (TS) 1 and at TS1+RR interval (TS3). Finally, the third block of images is acquired at TS2 and at TS2+*n*RR intervals (TS4). T1 estimation is optimized not only by the PD images, but also by the selection of the acquired saturation time images [27]. TS1 is set to 250 ms to acquire the shortest possible saturation image, while maintaining an adequate SNR. For most heart rates, TS2 is set to 500 ms to allow a TS4 similar to the native cardiac T1 (1500 ms at 3T). For high heart rates that make a 500-ms TS2 unfeasible, the sequence automatically computes the longest possible TS2 according to the RR interval derived from the heart rate defined by the user in the acquisition protocol. In this way, TS1 and TS2 ensure consistent sampling at the low saturation time area of the T1 relaxation curve for both low and high heart rates. TS3 is always acquired at the RR interval immediately after TS1; whereas, TS4 is calculated to allow acquisition as close as possible to 1500 ms; therefore, TS4 is acquired after *n* recovery heart beats computed according to the heart rate. In this way, the design ensures sampling close to the T1s of interest and that TS3 and TS4 are unconstrained by high heart rates, as shown in Table S1.

3D SACORA uses a spoiled linear turbo field echo (T1-TFE) acquisition (TR/TE/FA = 2.8ms/1.32ms/5°). The 3D sequence covers a volume of 322 × 322 × 60 mm with an in-plane resolution of 2.0 × 2.0 mm and a slice thickness of 10 mm reconstructed to 5 mm (12 slices). Composite radiofrequency (RF) pulses are used to achieve homogeneous saturation across different T1 values [28, 29]. Full image acquisition is accelerated using a k-space shutter and a spatial domain compressed SENSE factor of 4.5 to acquire a whole 3D volume in 2 independent TFE shots of echo train length of 76. Compressed SENSE technique solves an inverse problem with a sparsity constraint from data acquired using a balanced variable density incoherently undersampled k-space acquisition scheme with Poisson disc-style distribution. For reconstruction, the image is iteratively optimized according to the noise decorrelation, regularization, and coil sensitivities using a sparsity term based on wavelets transform [24, 30]. In this study, compressed SENSE was applied in the spatial domain in both phase-encoding directions with a fixed regularization parameter (maximum energy loss percentage equal to 30%) to keep a good balance between data consistency, sparsity constraint and noise reduction. The effect of different regularization parameters/denoising levels is shown in Fig. S1.

### Image processing

For 3D SACORA, T1 values are estimated by bounded Levenberg–Marquardt fitting using Bloch equations [5]. All sampling points are corrected according to the linear readout, and sampling points TS3 and TS4 are further corrected for the magnetization distortion caused by the readout effects of TS1 and TS2, respectively [31–33]. The T1 curves are fitted to a three-parameter (T1, α and M0) relaxation model following the signal model described in Fig. S2. Fitting algorithm and T1 map generation are integrated in the scanner reconstruction pipeline; therefore, the parametric T1 maps for the entire 3D volume are available almost immediately after acquisition.

### Validation

All studies were performed with a Philips Achieva 3T-Tx magnetic resonance imaging (MRI) scanner (Philips Healthcare, Best, the Netherlands) using a 32-channel cardiac coil. The acquisition parameters and T1 maps generation methodology were optimized for in vivo acquisition and validated with phantom experiments.

#### Phantom validation

The precision and accuracy of 3D SACORA were assessed in phantom experiments. The phantom consisted of eight tubes filled with distilled water and different concentrations of an MR contrast agent (Dotarem, Guerbet, Paris, France) selected to obtain T1s spanning the range from 355 ms to 1871 ms.

Reference T1 values were obtained using an inversion recovery spin-echo (IR-SE) sequence with a repetition time of 15 s and 15 inversion times ranging from 100 to 3500 ms. Other sequence parameters were slice thickness = 10 mm and TE = 18 ms. Each image was acquired in approximately 11 min.

To validate further 3D SACORA T1 estimation accuracy and precision, T1 values were also estimated using an in-house version of 3D SASHA [11]. This 3D SASHA variant used a linear spoiled T1-TFE readout without any respiratory trigger for phantom and in vivo experiments. In addition to the PD image, eight equally spaced saturation time images were acquired, starting from a minimum saturation time of 120 ms to a maximum saturation time dependent of the heart rate—709 ms in case of a heart rate of 60 bpm. PD recovery beats were computed automatically according to the heart rate so as to achieve a minimum T1 recovery time of 6 s. Other protocol characteristics were TR/TE/FA = 2.8ms/1.3ms/12°; slice thickness = 10 mm, reconstructed to 5 mm (12 slices); spatial domain compressed SENSE factor = 1.8; in-plane resolution = 2x2 mm; Cartesian radial k-space filling; and echo train length = 30. For a heart rate of 60 bpm, the acquisition time was approximately 3 minutes. The bounded three-parameter relaxation model-fitting algorithm and the T1 map generator were integrated in the scanner reconstruction pipeline.

T1 values (mean ± standard deviation) were measured from manually drawn regions of interest (ROIs) in the generated T1 maps.

Passing–Bablok regression and Bland–Altman plots were used to assess correlation and agreement between the three sequences.

To explore the sensitivity of the proposed methodology to heart rate, we acquired images with 3D SACORA and 3D SASHA at simulated heart rates ranging from 50 to 120 bpm. The estimated T1s were then compared against the T1 reference values obtained with IR-SE. As described before, both sequences automatically calculated the PD recovery beats, ensuring acquisition of fully recovered PD. Furthermore, the TS4 in 3D SACORA was automatically adjusted according to the heart rate to ensure acquisition within the range of T1 values of interest. The precision and accuracy of this approach were measured by the coefficient of variation:

$$\mathrm{C}\mathrm{V}=\frac{\mathrm{S}\mathrm{D}}{\mathrm{M}},$$

and relative error:

$$\mathrm{R}\mathrm{E}=\frac{{{M}_{\mathrm{p}\mathrm{r}\mathrm{o}\mathrm{p}\mathrm{o}\mathrm{s}\mathrm{e}\mathrm{d} \mathrm{M}\mathrm{e}\mathrm{t}\mathrm{h}\mathrm{o}\mathrm{d}}-M}_{\mathrm{r}\mathrm{e}\mathrm{f}\mathrm{e}\mathrm{r}\mathrm{e}\mathrm{n}\mathrm{c}\mathrm{e} \mathrm{M}\mathrm{e}\mathrm{t}\mathrm{h}\mathrm{o}\mathrm{d}}}{{M}_{\mathrm{r}\mathrm{e}\mathrm{f}\mathrm{e}\mathrm{r}\mathrm{e}\mathrm{n}\mathrm{c}\mathrm{e} \mathrm{M}\mathrm{e}\mathrm{t}\mathrm{h}\mathrm{o}\mathrm{d}}},$$

where SD is the standard deviation inside the ROI, M is the average value inside the ROI and reference method is the T1 map obtained from the IR-SE acquisition.

#### In vivo validation

Seven large white castrated male healthy pigs (mean weight, 40 kg) were scanned with 3D SACORA and 3D SASHA. Mean heart rate during 3D SACORA pre-contrast acquisition was 73 bpm (range, 55–83 bpm). In all pigs, images were acquired before and after administration of MR contrast agent. Scans were carried out under free-breathing conditions. No respiratory navigator approach was implemented because abdominal respiration in pigs produces little chest movement under free-breathing conditions.

The study protocol was approved by the local institutional Animal Research Committee and conducted in accordance with recommendations of the Guide for the Care and Use of Laboratory Animals.

Bulls-eye plots of T1 and coefficient of variation were generated from 3D SACORA and 3D SASHA data according to the American Heart Association (AHA) standard 17-segment model of the LV. For both techniques, the central segment was used to report blood values. The segment values used were mean values of the seven pigs. These plots were used to provide information about myocardium homogeneity and give a 3D perspective on the estimated T1s.

For 3D SACORA and 3D SASHA, septal T1 values (mean ± standard deviation) were measured from ROIs manually drawn on the septal myocardium. These measurements were used to compare the septal T1 values and coefficient of variation obtained by both sequences.

## Results

### Phantom validation

**Fig. 2:**
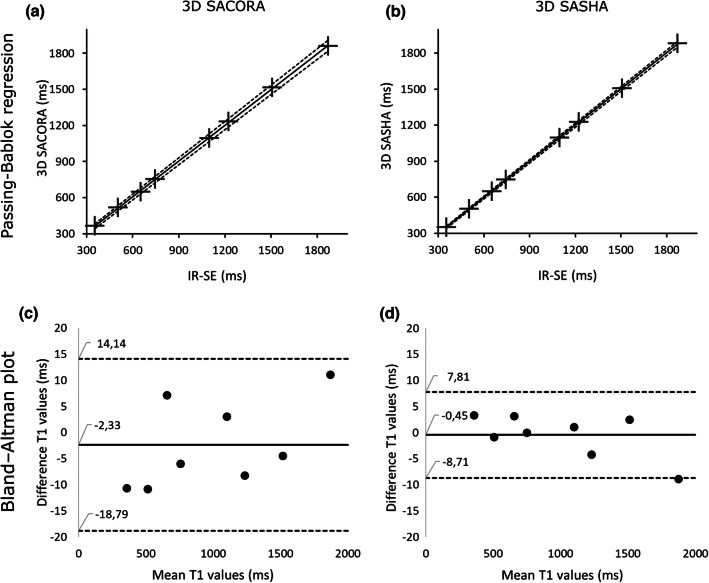
3D SACORA and 3D SASHA results from phantom experiments validated against IR-SE.** a, b** Passing–Bablok regressions of 3D SACORA and 3D SASHA. **c**, **d** Bland–Altman plots of 3D SACORA and 3D SASHA

The Passing–Bablok regression plots presented in Fig. 2 show good correlation and no significant bias between methods. The 3D SACORA plot (Fig. 2a) has a slope of 0.99 (95% confidence interval [CI] 0.98, 1.01) and an intercept of 12.90 ms (95%CI  − 6.19, 23.02 ms); whereas, the 3D SASHA plot (Fig. 2b) has a slope of 1.005 (95%CI 0.997, 1.013) and an intercept of  − 4.62 ms (95%Cl  − 11.60, 2.47 ms).

The Bland–Altman plots in Fig. 2 compare reference T1s with T1s estimated by 3D SACORA and 3D SASHA. These plots show good agreement between both methods and IR-SE. For 3D SACORA (Fig. 2c), the bias was  − 2.3 ms (95%CI  − 18.79, 14.14 ms). For 3D SASHA (Fig. 2d), the bias was  − 0.45 ms (95%CI  − 8.71, 7.81 ms).

**Fig. 3:**
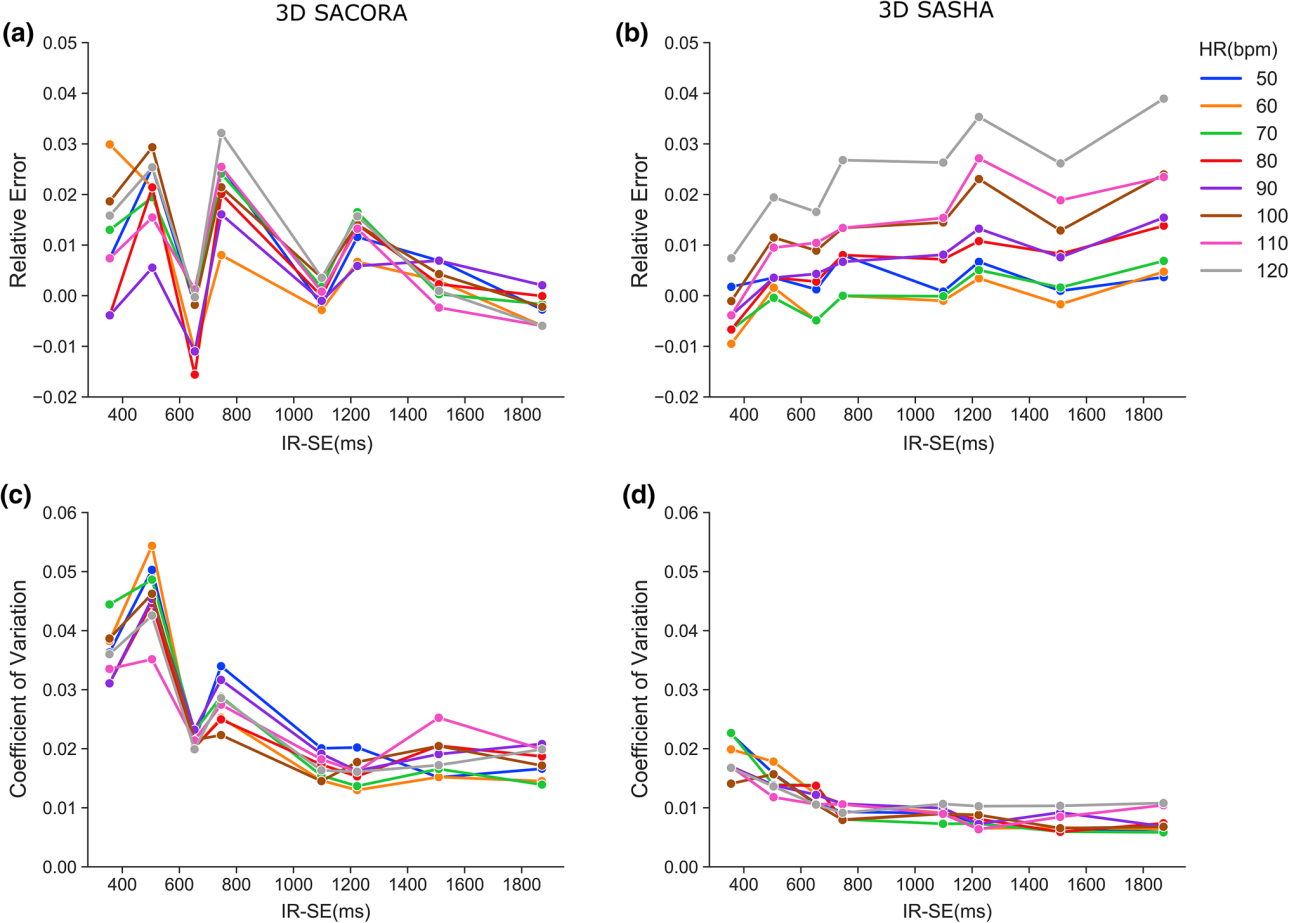
Phantom experiments assessing the HR dependency of 3D SACORA and 3D SASHA. **a**, **b** Relative error of 3D SACORA and 3D SASHA at different HRs. **c**, **d** Coefficient of variation of 3D SACORA and 3D SASHA at different HRs

The relative error at different heart rates ranged from  − 0.016 to 0.032 for 3D SACORA (mean, 0.008; standard deviation, 0.011); for 3D SASHA the range was  − 0.0095 to 0.039 (mean, 0.008; standard deviation, 0.01) (Fig. 3a, b). 3D SACORA showed less dependence on heart rate, particularly for pre-contrast cardiac T1 values at 3T; estimated T1 values with 3D SASHA tended to increase with higher heart rate; whereas, 3D SACORA T1s seemed to be relatively independent of heart rate. Regarding accuracy, both sequences performed equally well.

The coefficient of variation at different heart rates ranged from 0.013 to 0.054 for 3D SACORA (mean, 0.025; standard deviation, 0.011); for 3D SASHA, the range was 0.006–0.023 (mean, 0.011; standard deviation, 0.004) (Fig. 3c, d). Although precision was acceptable with both sequences, 3D SASHA performed slightly better, particularly on short T1s. The coefficient of variation appeared to be independent of heart rate in both sequences.

### In vivo validation

Scans of the seven pigs were performed with 3D SACORA and 3D SASHA before and after MR contrast agent administration. 3D SACORA acquired the 3D T1 map in 15 heart beats (heart rate, 60 bpm). For both sequences, the final T1 parametric maps were obtained from the scanner shortly after acquisition. Reconstruction, fitting, and image generation took approximately 20s.

**Fig. 4:**
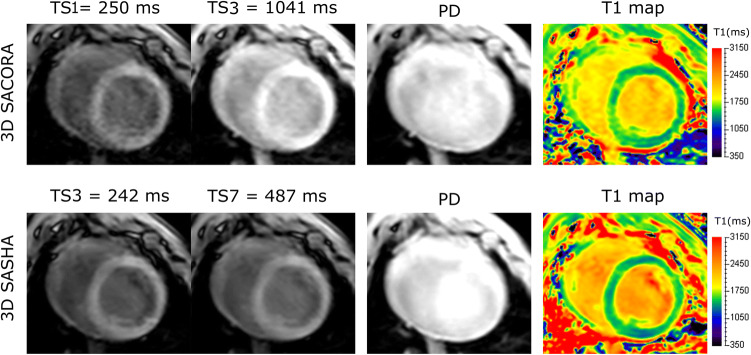
Representative images acquired from pig 6 with 3D SACORA and 3D SASHA (two T1-weighted images and a PD image) and the corresponding T1 maps

Four images from the same animal for each sequence are shown in Fig. 4: two T1-weighted images, a PD image, and the corresponding T1 map. All T1-weighted images showed good contrast and quality. The TS1 image from 3D SACORA presented some noise, likely due to the short saturation time and relatively high compressed SENSE factor (4.5). No contrast was visible between myocardium and blood pool in the PD images, as expected. The T1 maps correctly represented the information contained in the T1-weighted images.

**Fig. 5:**
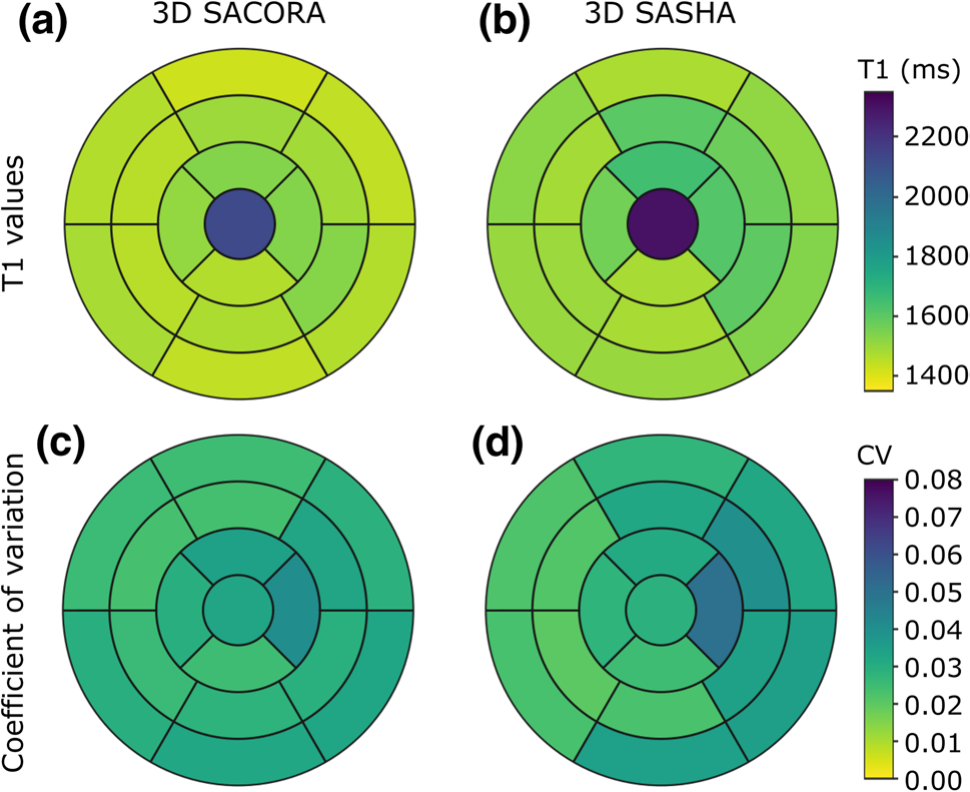
Bulls-eye plots of T1 and coefficient of variation (CV) for the whole left-ventricle myocardium with 3D SACORA and 3D SASHA. The central segment corresponds to the blood. **a,** **b** Bulls-eye plots of mean T1 values for both sequences. **c**, **d** Bulls-eye plots of mean CV for both sequences

Bulls-eye plots were calculated for 3D SACORA and 3D SASHA according to the AHA LV model (Fig. 5). Bulls-eye plots of mean T1 values (Fig. 5a, b) showed acceptable homogeneity across the LV myocardium for both sequences. The mean T1 for the whole LV myocardium was 1480 ± 33 ms with 3D SACORA and 1539 ± 54 ms with 3D SASHA. For the blood pool, the mean T1 values were 2126 ± 104 ms and 2306 ± 93 ms, respectively. The difference between the two sequences in mean whole LV myocardium T1 was mainly due to T1 measurements in the lateral and anterior segments. The bulls-eye plots for coefficient of variation (Fig. 5c, d) showed good precision in the measurement of T1 values in myocardium and blood for both sequences. The mean coefficients of variation in 3D SACORA were 0.029 ± 0.005 in the whole LV myocardium and 0.033 ± 0.006 in the blood pool; the corresponding values for 3D SASHA were 0.030 ± 0.008 and 0.029 ± 0.005.

**Fig. 6:**
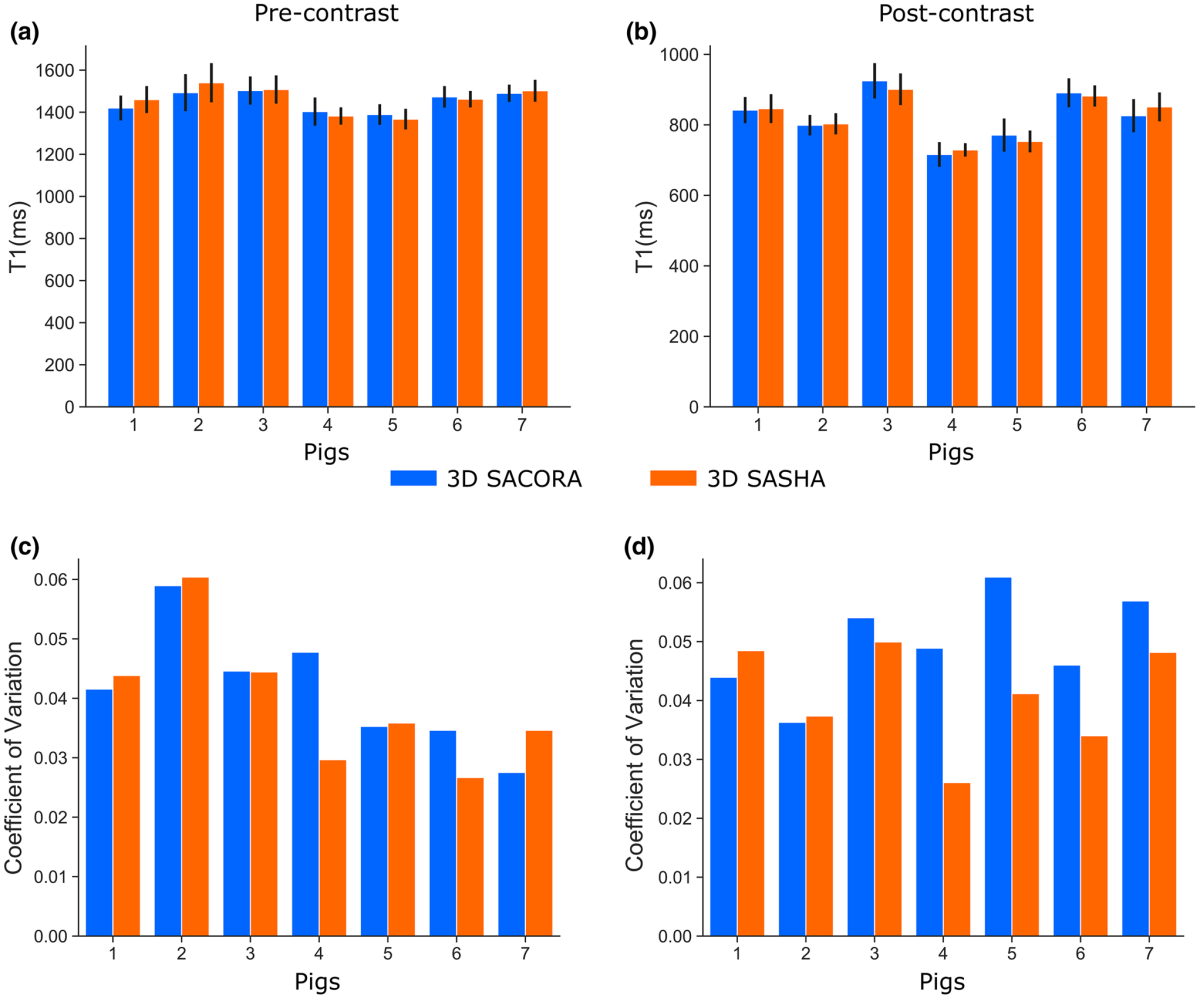
Septal T1 measurements (pre-contrast and post-contrast) obtained from all pigs with 3D SACORA and 3D SASHA. **a** Pre-contrast septal T1 and standard deviation for both sequences. **b** Post-contrast septal T1 and standard deviation for both sequences. **c** Septal coefficient of variation of pre-contrast acquisitions for both sequences. **d** Septal coefficient of variation of post-contrast acquisitions for both sequences

Septal T1s and coefficients of variation were measured with 3D SACORA and 3D SASHA in all seven pigs before and after administration of MR contrast agent (Fig. 6). Native and post-contrast T1 (Fig. 6a, b) did not differ between the two sequences, confirming good accuracy and precision. Mean septal native and post-contrast T1s measured with 3D SACORA were 1453 ± 44 ms and 824 ± 66 ms, respectively. For 3D SASHA, the mean septal native T1 was 1460 ± 60 ms and the mean septal post-contrast T1 was 824 ± 60 ms. The coefficient of variation plots (Fig. 6c, d) showed acceptable precision for both sequences in all animals. The mean coefficient of variation for native septal T1 was 0.041 ± 0.010 for 3D SACORA and 0.039 ± 0.010 for 3D SASHA. The post-contrast values were 0.050 ± 0.008 and 0.041 ± 0.008, respectively.

**Fig. 7:**
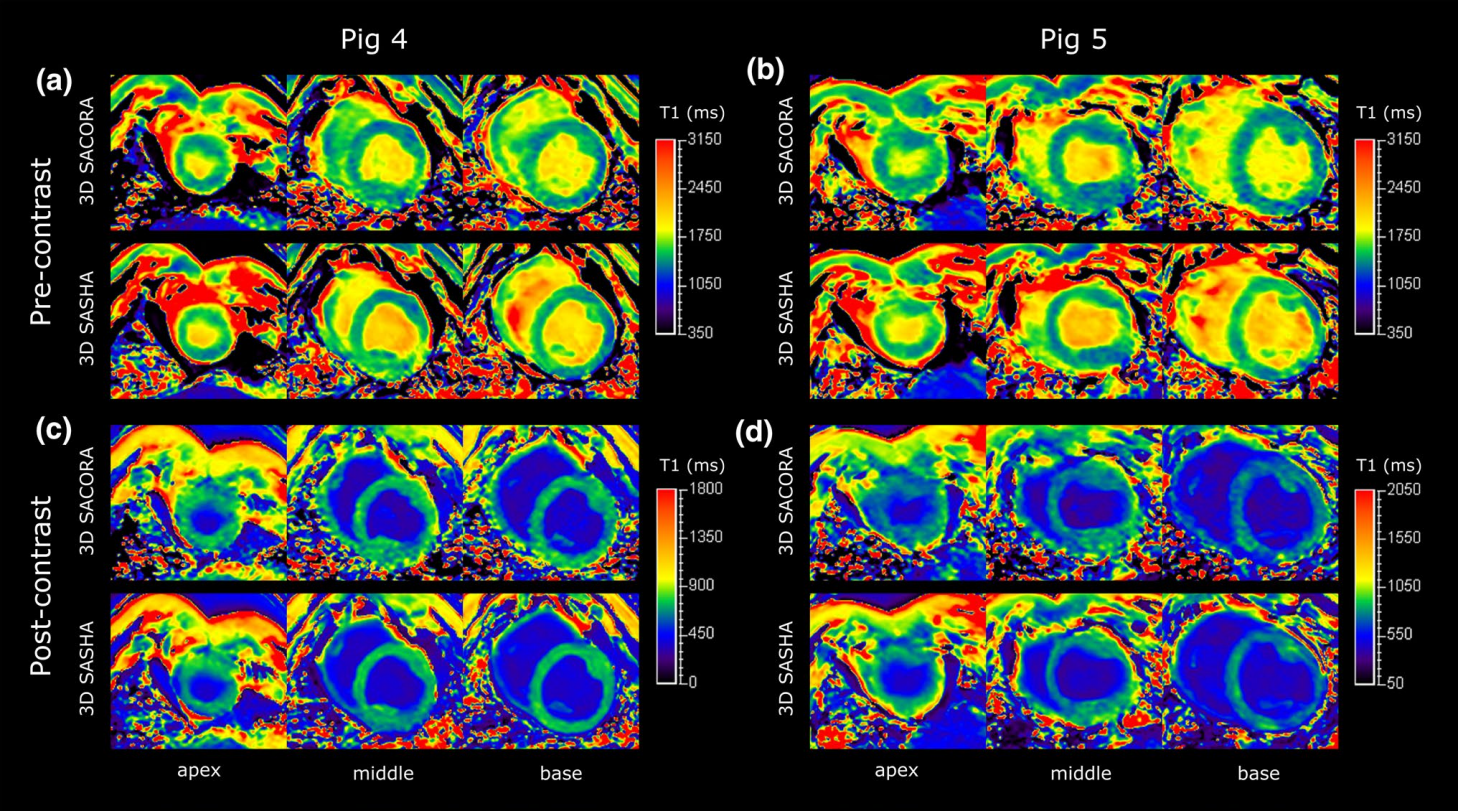
Pre-contrast and post-contrast T1 maps of three slices (apex, middle, base) acquired with 3D SACORA and 3D SASHA from pigs 4 and 5. **a**, **b** Pre-contrast images from pigs 4 and 5. **c**, **d** Post-contrast images from pigs 4 and 5

Representative pre-contrast and post-contrast T1 maps of three slices (apex, middle, base) acquired with 3D SACORA and 3D SASHA in two pigs are shown in Fig. 7. 3D SACORA images showed good contrast, acceptable homogeneity, and were comparable to corresponding 3D SASHA images, despite the shorter acquisition time (15s vs. 188s, for a heart rate of 60 bpm).

## Discussion

3D SACORA (3D saturation recovery compressed SENSE rapid acquisition) was developed as a new 3D T1 mapping sequence to speed up T1 mapping acquisition of the whole heart. The proposed sequence was validated in phantoms against the gold standard technique (IR-SE) and in vivo against the previously proposed sequence 3D SASHA.

3D SACORA successfully acquired a whole-heart 3D T1 map in a single breath-hold at 3T, estimating T1 values in agreement with those obtained with the IR-SE and 3D SASHA sequences. Thus, 3D SACORA’s use of 5 saturation times for T1 fitting as well as k-space under-sampling via compressed SENSE enabled very short acquisition times (15s, for a heart rate of 60 bpm) without significantly compromising T1 estimation accuracy or image quality.

3D saturation recovery T1 mapping sequences have been developed recently because they do not require full longitudinal magnetization recovery and produce highly accurate T1 values. Acquisition times with these 3D sequences are, however, much longer than 20 s, and scans, therefore, cannot be performed under single breath-hold conditions. 3D SACORA was optimized to keep scan time shorter than 20 s without compromising T1 estimation accuracy, image quality, or LV coverage. Conditions established for this optimization included (i) allowing enough time for the PD to achieve full magnetization recovery between readouts, (ii) limiting the number of turbo field echo shots as much as possible, (iii) optimizing trade-off between readout length and the compressed SENSE factor, (iv) acquiring T1-weighted images with enough SNR for proper application of compressed SENSE, and (v) acquiring a T1-weighted image with a saturation time as close as possible to the native cardiac T1 values at 3T.

3D SACORA is able to acquire a 3D T1 map in 15 heart beats (heart rate, 60 bpm) at 3T. The main constraint for acquiring T1 maps with a short scan time at 3T is the recovery beats required by the proton density. To mitigate this, 3D SACORA was designed to have just two readout shots. This was achieved by combining a shot length of ~210 ms with a compressed SENSE factor of 4.5, providing good image quality without major deterioration or blurring. A compressed SENSE factor of 4.5 guarantees good T1 estimation accuracy and precision, with comparable results to lower compressed SENSE factors, as shown in Fig. S3. A shot length of ~210 ms is similar to the conventional 2D MOLLI shot length of ~190 ms [4] and shorter than the diastolic time length at very high heart rates (250 ± 59 ms at 128 ± 22 bpm) [34]. The PD readouts were separated by 6 s to guarantee full magnetization recovery. 3D SACORA acquires two T1-weighted images with long saturation times to enhance fitting quality for relevant cardiac T1 values at 3T. In addition, these images are ideal for applying compressed SENSE due to their high SNR; whereas, T1-weighted images with shorter saturation times tend to be noisier, as shown in Fig. 4.

3D SACORA and our own implementation of 3D SASHA were validated in phantom experiments. 3D SASHA is the reference sequence for in vivo experiments in this study and has an acquisition time roughly 13 times longer than 3D SACORA. The phantom results show that 3D SACORA and 3D SASHA acquired 3D T1 maps with high accuracy and precision and in good agreement with IR-SE measurements. However, slight differences were found between 3D SACORA and 3D SASHA in specific cases (Fig. 3). First, 3D SASHA is more precise than 3D SACORA for short T1s, due to the denser sampling of short saturation time T1-weighted images present in 3D SASHA. Second, 3D SACORA T1 estimation is, by design, less heart-rate sensitive than 3D SASHA. Heart-rate sensitivity in 3D SASHA acquisition is due to its lack of long saturation images, which can undermine T1 estimation quality, especially at high heart rates. Thus, the 3D SACORA sampling strategy was validated over a wide range of heart rates (50 bpm–120 bpm). For heart rates outside this range, the sequence keeps acquiring saturation times at the low saturation time area of the T1 relaxation curve and close to the T1s of interest, as shown in Table S1. This sampling strategy of the saturation times makes 3D SACORA robust for a very wide range of heart rates.

The pig heart is an established model in cardiology due to its similarity to the human heart [35, 36]. In this study, we acquire in vivo data before and after contrast administration. The image quality in 3D SACORA was close to that obtained with 3D SASHA despite the much shorter acquisition time. All septal T1 measurements were similar in the two sequences (Fig. 6a, b), despite the differences in sequence design and protocol. Furthermore, the mean septal pre-contrast T1 of 1453 ms estimated by 3D SACORA is in good agreement with published saturation recovery T1 measurements in pigs at 3T [29]. There was a slight discrepancy between 3D SACORA and 3D SASHA in T1s measured in the lateral and anterior segments of the myocardium (Fig. 5a, b), probably caused by movement artifacts, which were more frequent in 3D SASHA due to the longer acquisition time.

In pigs, cardiac acquisitions performed in free-breathing are free of major respiratory artifacts [36, 37]. This was crucial for the successful comparison of 3D SACORA with 3D SASHA in a model without respiratory motion compensation (e.g., respiratory navigator [12] or motion correction [38]) or breath-hold acquisition. Nevertheless, the results might be improved by taking appropriate measures to minimize respiratory artifacts. For example, T1 map quality could be improved by reducing respiration-induced motion using registration approaches such as non-rigid image registration [39].

In this study, we used an in-house version of 3D SASHA for 3T [11]. As the reference sequence for the in vivo experiments, the sampling strategy of the saturation times was similar to conventional 3D SASHA to acquire gold standard in vivo T1 values. In addition, 3D SASHA was implemented with a shorter shot length than 3D SACORA, minimizing the effect of cardiac motion and reducing partial volume averaging, which are especially relevant for high heart rates. This sequence was successfully validated against IR-SE with phantom experiments, and the in vivo results were in good agreement with published saturation recovery T1 measurements at 3T [12, 29].

Despite the good performance of 3D SACORA in post-contrast imaging, the sequence was primarily designed for pre-contrast imaging. Although post-contrast T1 values obtained with the proposed technique do not differ significantly from those obtained with 3D SASHA, the accuracy and precision of short T1 could be improved by increasing the number of short saturation images. The additional acquisition time could be compensated by decreasing the number of PD recovery beats.

One of the main limitations of this feasibility study is the lack of in vivo human data. Nevertheless, pig model is a well-stablished model for cardiac research and in vivo pig acquisitions can be performed under free-breathing conditions without significantly decreasing image quality [35–37]. Additionally, feasibility studies of new cardiac acquisitions have been successfully performed on pigs [40–43]. A clinical study will be required to evaluate the performance of 3D SACORA (single breath-hold of 15s, for a heart rate of 60 bpm) in patients with ischemic and non-ischemic cardiomyopathies.

In conclusion, the proposed 3D SACORA sequence acquired pre-contrast and post-contrast T1 maps of the whole heart with good accuracy, precision, and image quality for LV analysis at 3T. The sequence was optimized for speed and can acquire a 3D T1map in 15 heart beats for a heart rate of 60 bpm.

## Electronic supplementary material

Below is the link to the electronic supplementary material.

Supplementary file1 (DOCX 1171 kb)

## Abbreviations

CMR: Cardiovascular magnetic resonance
T1: Longitudinal relaxation time
2D: Two-dimensional
MOLLI: Modified Look-Locker inversion recovery
SSFP: Steady-state free precession
SASHA: Saturation recovery single-shot acquisition
SNR: Signal–noise ratio
MLLSR: Modified Look–Locker acquisition using saturation recovery
SAPPHIRE: Saturation pulse-prepared heart rate independent inversion recovery
3D: Three-dimensional
LV: Left ventricle
FLASH: Fast low angle shot
T2: Transverse relaxation time
SENSE: Sensitivity encoding
SACORA: Saturation recovery compressed SENSE rapid acquisition
HR: Heart rate
MRI: Magnetic resonance imaging
PD: Proton density
TS: Saturation time
TFE: Turbo field echo
TR: Repetition time
TE: Echo time
FA: Flip angle
RF: Radiofrequency
IR-SE: Inversion recovery spin-echo
ROI: Region of interest
CV: Coefficient of variation
MR: Magnetic resonance
AHA: American heart association
Cl: Confidence interval
