# Supplementary material for: Single breath-hold saturation recovery 3D cardiac T1 mapping via compressed SENSE at 3T
Source: MAGMA. 2020 May 14;33(6):865–76. doi: 10.1007/s10334-020-00848-2 (PMC7669807; doi:10.1007/s10334-020-00848-2)

**Single breath-hold saturation-recovery 3D cardiac T1 mapping via compressed SENSE at 3T**

Tiago Ferreira da Silva^1,2^, Carlos Galan-Arriola^2,3^, Paula Montesinos^1^, Gonzalo Javier López-Martín^2^, Manuel Desco^2,3,4,5^, Valentín Fuster^2,6^, Borja Ibáñez*^2,3,7^, Javier Sanchez-Gonzalez*^1^

**Author affiliation**: ^1^Philips Healthcare Iberia, Madrid, Spain; ^2^Centro Nacional de Investigaciones Cardiovasculares Carlos III (CNIC), Madrid, Spain; ^3^CIBER de Enfermedades Cardiovasculares (CIBERCV), Madrid, Spain; ^4^Departamento de Bioingeniería e Ingeniería Aerospacial, Universidad Carlos III, Madrid, Spain; ^5^Medicina y Cirugía Experimental, Instituto de Investigacion Sanitaria Gregorio Marañón, Madrid, Spain; ^6^The Zena and Michael A. Wiener CVI, Icahn School of Medicine at Mount Sinai, New York, USA; ^7^IIS-Fundación Jiménez Díaz University Hospital, Madrid, Spain.

**e-mails**: Tiago.Ferreira@philips.com, carlos.galan@cnic.es, Paula.Montesinos@philips.com, gonzalojavier.lopez@cnic.es, desco@hggm.es, vfuster@cnic.es, bibanez@cnic.es, Javier.Sanchez.Gonzalez@philips.com

***Corresponding author:**

**Javier Sánchez-González, PhD**. MR Clinical Scientist Philips Healthcare Iberia. C\María Portugal 1, 29050 Madrid (Spain) Email: [Javier.Sanchez.Gonzalez@philips.com](mailto:Javier.Sanchez.Gonzalez@philips.com)

**Or**

**Borja Ibanez, MD PhD**. Director, Translational Laboratory for Cardiovascular Imaging and Therapy. Centro Nacional de Investigaciones Cardiovasculares Carlos III (CNIC). Melchor Fernandez Almagro, 3. 28029 Madrid (Spain). Email: [bibanez@cnic.es](mailto:bibanez@cnic.es)**Supplementary material**

**Table S1**: Table with the sampling strategy of the saturation times of 3D SACORA for different heart rates (40 – 120 bpm). The saturation times change with the heart rate to cover the low saturation time area of the T1 relaxation curve and to ensure sampling close to the T1s of interest.


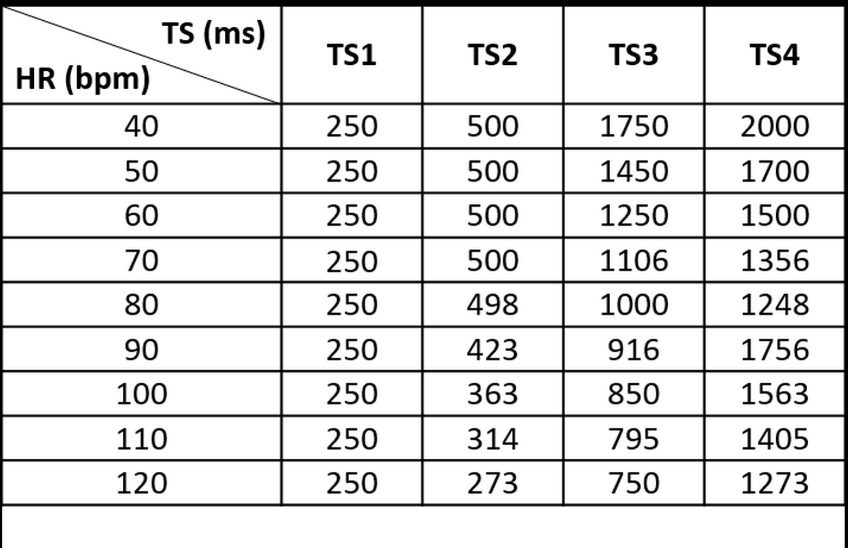


**Figure S1**: Figure showing the effect of different regularisation parameters levels on the estimated T1 maps with two different regularization factors: a) medium (maximum energy loss percentage, 15%) and b) strong (maximum energy loss percentage, 30%).


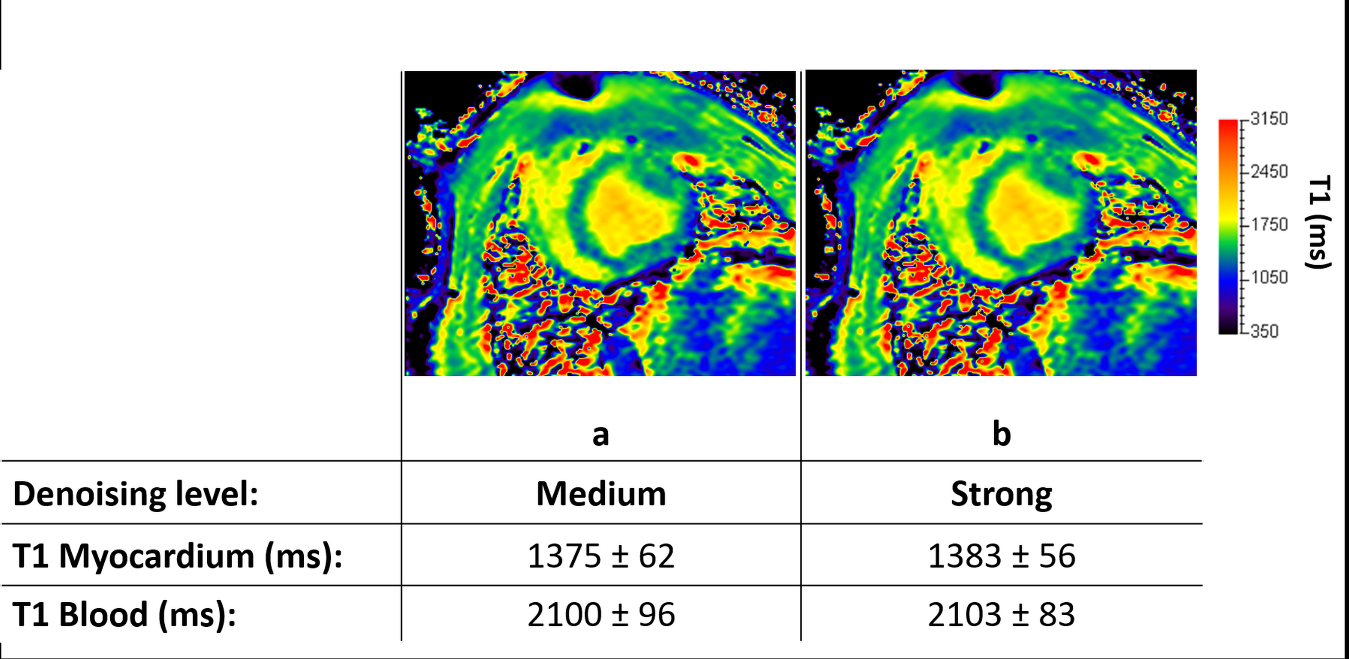


**Figure S2**: Schematic representation of the magnetization evolution during a 3D SACORA acquisition. M1, M2, M3, M4, and M5 take into account freely signal recovery after saturation pulse as well as the signal evolution derived from excitation pulses during data acquisition for a single pair of saturation times (TS2 and TS4). T1* and M0* represent the apparent T1 and magnetization during signal readout using spoiled turbo field echo acquisition. TR = repetition time, θ = flip angle, α = saturation pulse efficiency.


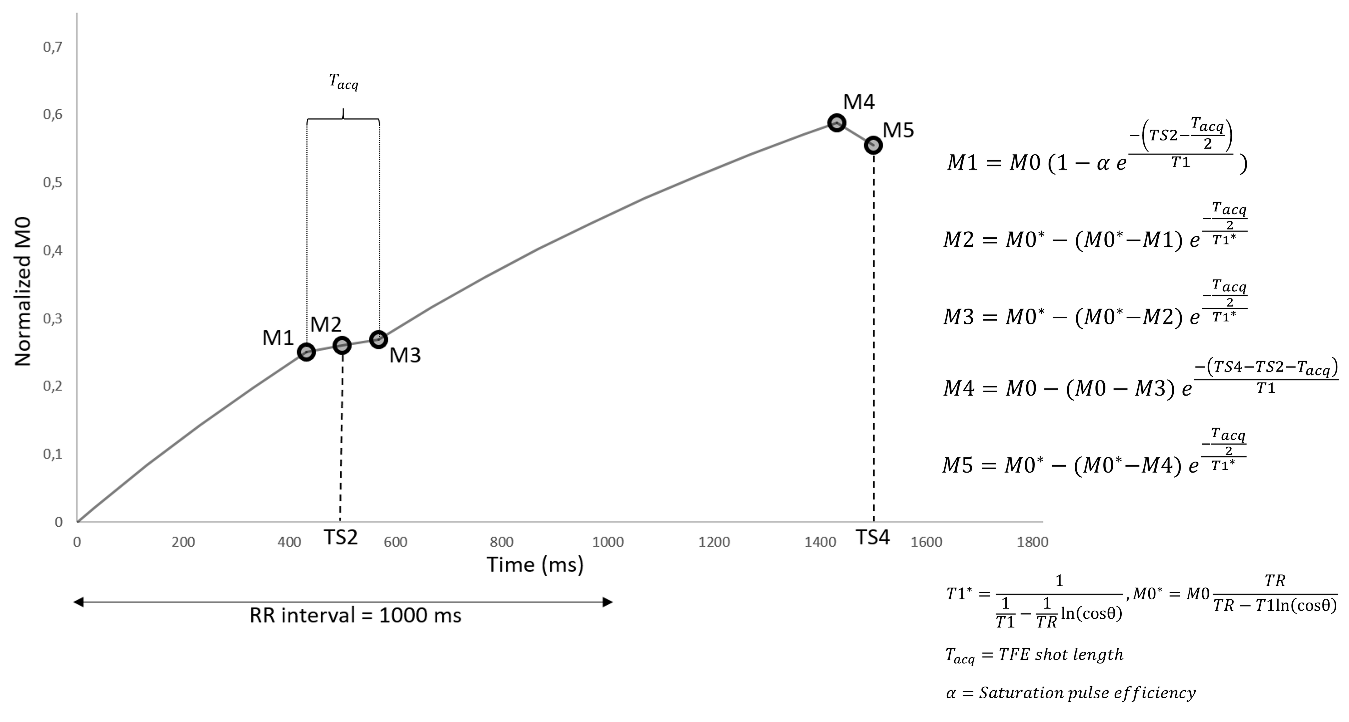


**Figure S3**: Phantom acquisitions with a simulated heart rate of 60 bpm, showing a) the accuracy and b) precision of 3D SACORA for different compressed SENSE factors (1.5, 3 and 4.5).


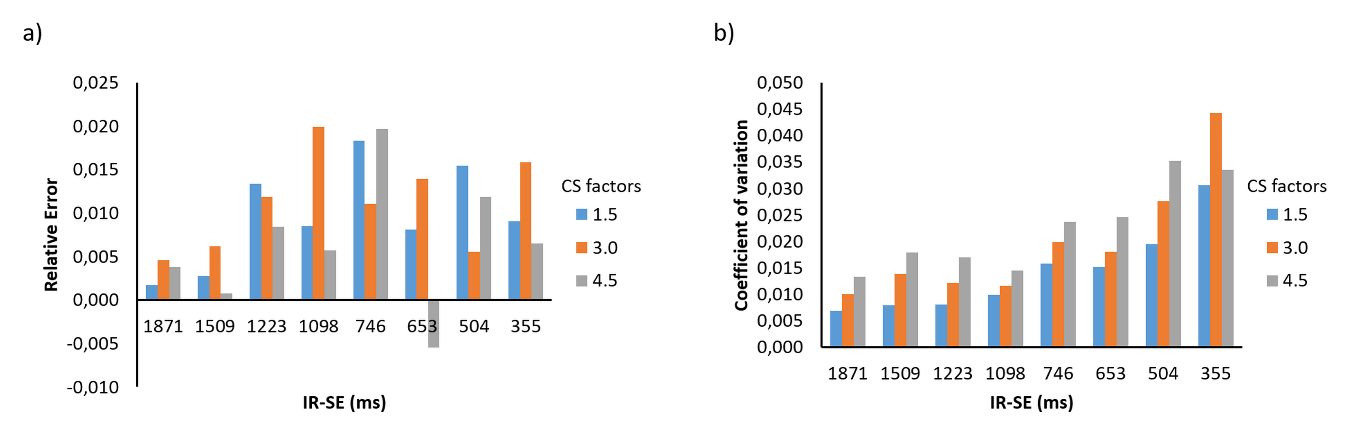

Supplement: Supplementary file 1 — Supplementary file1 (DOCX 1171 kb) [file 10334_2020_848_MOESM1_ESM.docx]
